# Acute Inhalant-Induced Atrial Fibrillation With Severe Hypocalcemia: A Case Report and Review of the Pathophysiology

**DOI:** 10.7759/cureus.19897

**Published:** 2021-11-25

**Authors:** Nicholas George, Brian Chin, Arianna S Neeki, Fanglong Dong, Michael M Neeki

**Affiliations:** 1 Department of Emergency Medicine, Arrowhead Regional Medical Center, Colton, USA; 2 Department of Emergency Medicine, California University of Science and Medicine, Colton, USA

**Keywords:** toxicology, emergency medicine, hypocalcemia, atrial fibrillation, difluoroethane, illicit substances, inhalant abuse

## Abstract

The recreational use of inhalants is associated with various detrimental health effects ranging from inebriation to cardiac arrest. It also presents a challenging clinical problem as the diagnosis is made by the presentation and patient’s history, which is often difficult to obtain in an intoxicated or obtunded individual. The incidence of inhalant use is relatively high. National surveys have reported that nearly 21.7 million Americans aged 12 and older have used inhaled substances at least once in their lives. There is no reversal agent or antidote for inhalants and supportive care is generally recommended. We present a case of a young patient presenting with acute inhalant toxicity accompanied by atrial fibrillation with a rapid ventricular response and severe hypocalcemia.

## Introduction

Recreational use of inhalants or “inhalant use” is the intentional inhalation of vapors through various methods to achieve intoxication. Inhalants are toxic and even a single use can be fatal with both acute and chronic deleterious effects on multiple organ systems. Despite tight regulations by the states and federal regulatory agencies on the distribution of these products, they continue to be available in many households and can be readily obtained over the counter. Inhalants are frequently among the first recreational drugs used by youths due to their low cost and wide availability [1]. The National Institute of Drug Abuse (NIDA) reported that nearly 21.7 million Americans aged 12 and older have used inhalant substances at least once in their lives [2]. The National Survey on Drug Use and Health reported that inhalants use increased from 1.7 million people in 2016 to 2.1 million people in 2019 [3]. The same survey also reported that 63% of inhalant users were younger than 25 years old [3]. Furthermore, NIDA reported the percentage of eighth-grade students who used inhalants in their lifetime increased from 9.5% in 2019 to 12.6% in 2020, while the percentage of 10th-grade students who used inhalants in their lifetime, increased from 6.8% in 2019 to 7.4% in 2020 [4]. These data suggest that illicit inhalants use is a significant problem in the United States, notably in adolescents and young adults, and the incidence appears to be increasing.

Inhalants consist of multiple toxic components and the most commonly used products contain halogenated hydrocarbons such as 1,1-difluoroethane (DFE) and its various isomers [5]. The chemical components are toxic and can induce immediate and delayed side effects, including asphyxia, anoxia, aspiration events, and trauma [6]. Inhalant use has been reported to be associated with various adverse cardiovascular effects, including bradycardia induced by vagal stimulation, reduced sinoatrial node automaticity with atrioventricular block, and myocardial ischemia and infarction [7]. Chronic inhalant use can lead to cardiomyopathies such as myocarditis, which may lead to further cardiovascular complications including fatal arrhythmias in the acute setting [8]. This case report describes a presentation of a young patient with acute inhalant toxicity accompanied by cardiac arrhythmia and severe hypocalcemia in the emergency department. This study was approved by the institutional review board (IRB) at Arrowhead Regional Medical Center with the IRB approval number 21-12.

## Case presentation

A 23-year-old male was transported by the emergency medical services (EMS) in the early hours of the morning for altered mental status. His girlfriend called the EMS as they had been at a gathering with friends earlier that evening. He allegedly had taken a few oxycodone tablets and later in the evening was seen unresponsive with a can of empty “Dust-Off” spray near him. Suspecting opioid overdose, the EMS crew administered 4 mg of intranasal naloxone with no significant improvement.

On arrival at the emergency department (ED), the patient was non-verbal. His past medical history was significant for major depression. He was not currently taking any medications. On presentation, the patient was noted to have blood pressure (BP) of 156/110 millimeters of mercury (mmHg), heart rate (HR) of 140 beats per minute (BPM), with an irregularly irregular rhythm, observed on the cardiac monitor, temporal temperature of 96.5 degrees Fahrenheit, respiratory rate of 25 breaths per minute with an oxygen saturation of 97% on room air. His Glasgow Coma Scale (GCS) was 7 (E1V2M4). His pupils were 3 mm bilaterally, equal and reactive to light, and accommodating. His mucous membranes were dry. He was tachycardic with irregularly irregular rhythm without any murmurs. His skin was warm and dry. The rest of his initial physical exam did not reveal any abnormalities.

**Figure 1:**
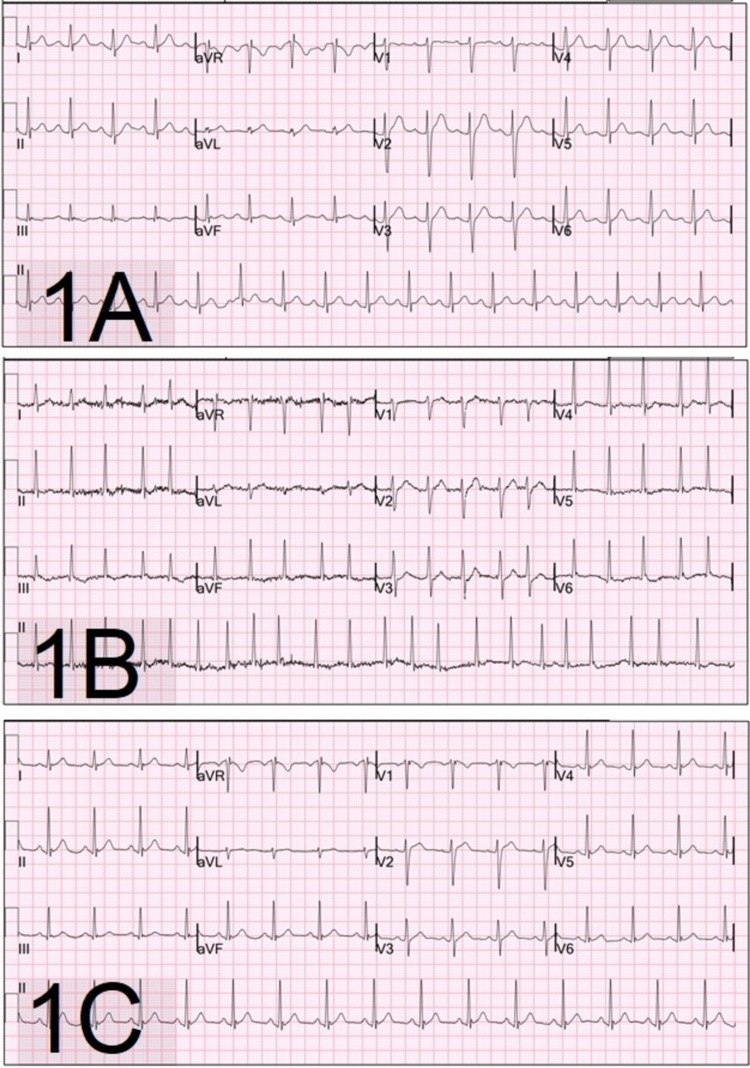
(A) EKG three years prior to presentation demonstrating sinus rhythm. (B) EKG at time of presentation demonstrating atrial fibrillation with rapid ventricular response. (C) EKG done four hours after presentation demonstrating return to normal sinus rhythm.

Point-of-care glucose was 156 milligrams per deciliter (mg/dL). An electrocardiogram (EKG) confirmed the suspected diagnosis of atrial fibrillation with a rapid ventricular response at a rate of 134 beats per minute with a corrected QT interval within the normal range (Figure 1B). An EKG in his electronic medical record documented three years prior to this visit revealed sinus rhythm without any notable abnormalities (Figure 1A). Hematology revealed leukocytosis of 19.6 thousand cells per microliter. Chemistry was notable for carbon dioxide level of 15 millimoles per liter, the total serum calcium level of 5.9 mg/dL which corrected to 6.5 mg/dL for hypoalbuminemia. Transaminitis was noted with elevated levels of aspartate aminotransferase to 423 units per liter (U/L) and alanine aminotransferase to 360 U/L. Laboratory workup otherwise revealed normal electrolytes including magnesium, normal blood urea nitrogen (BUN) and creatinine, creatine kinase, troponin, thyroid studies. Additionally, this patient had a negative Novel Coronavirus (Covid) polymerase chain reaction (PCR) test, normal blood alcohol level, negative acetaminophen, negative salicylate levels, and negative urine drug screen. A computed tomography scan of the head without contrast revealed no acute intracranial abnormality.

The patient was initially given 20 mg of intravenous (IV) diltiazem and 1 liter of IV lactated Ringer’s solution prior to resultant laboratory studies. His HR decreased to 110 BPM and systolic BP transiently dropped but remained about 100 mmHg for the duration of the care. He quickly became more lucid to GCS 15 and admitted to inhaling an over-the-counter keyboard cleaner. Once the hypocalcemia was noted, he was administered 1 gram of IV calcium gluconate. Within three hours of the presentations to ED and while under ED supportive care, he spontaneously converted to normal sinus rhythm with variable rates ranging from 60 to 90 BPM (Figure 1C). He was admitted to the telemetry unit for monitoring. During his stay in the hospital, his electrolytes normalized and his liver enzymes level remained elevated. No further arrhythmias had been noted on continuous telemetry monitoring and he did not require oxygen support or any further medications.

## Discussion

In the emergency clinical setting, the diagnosis of inhalant-induced cardiac arrhythmia can be very difficult without a reliable history. Currently, there is no readily and widely available laboratory test for acute inhalant toxicity. The use of current specific laboratory tests, such as urinary trichloroethanol and chlorinated hydrocarbons, is limited by their high costs, variable turnaround time, and scant availability [9]. While the presentation of patients may vary, acute inhalant toxicity has generally been associated with central nervous system (CNS) depression, metabolic acidosis, and arrhythmia, as the patient presented in this case [10]. In addition, inhalants can cause anoxia, respiratory depression, and vagal inhibition, all of which also contribute to CNS depression with variable duration [6]. There is no known reversal agent for inhalant toxicities and the treatment is primarily supportive care [5].

The patient in this case presented with atrial fibrillation, which is a common arrhythmia. Atrial fibrillation may occur in the absence of known cardiac structural or electrophysiological abnormalities as a trigger that initiates reentrant waves in a vulnerable atrial substrate [11]. The mechanism of arrhythmias induced by inhalants has been postulated as hydrocarbons “sensitizing” the myocardium to epinephrine by stabilizing myocardial cell membranes to repolarization, resulting in blocking electrical impulse conductions which increases the risk of arrhythmia [6]. Furthermore, as the volatile inhalant dissolves into cell membranes with high lipid affinity, the risk of arrhythmia can be prolonged up to a few hours even following improvement or resolution of the patient’s acute intoxication state [6]. Therefore, the risk of developing arrhythmias following inhalant use is high, even in the absence of prior heart disease.

The most feared complication was first described by Bass in 1970 and it is termed “sudden sniffing death,” which is reported to be the cause of more than half of all deaths due to inhalant toxicity [12]. This “sudden sniffing death” phenomenon may occur hours after the initial inhalation due to any endogenous increases in epinephrine, leading to the development of ventricular fibrillation which is often resistant to defibrillation [13,14]. The treatment consists of avoiding further exogenous catecholamines such as epinephrine, and counteracting endogenous catecholamines with a beta-blocker such as esmolol [15,16]. The true incidence and prevalence of atrial fibrillation secondary to inhalant use are unknown as few cases have been reported.

This patient also presented with severe hypocalcemia. Inhalants contain various fluorinated ethanes which have been shown to cause hypocalcemia. Fluorinated ethanes are absorbed and then metabolized at the hydrogen-carbon bonds to fluoroacetate via an aldehyde or acyl fluoride. Fluoroacetate then forms a complex with coenzyme A and is metabolized into fluorocitrate which accumulates and chelates serum calcium. This chelation process by citrate is the primary cause of hypocalcemia from DFE inhalation [17]. In animal studies, rats exposed to DFE demonstrated increases in serum citrate levels up to fivefold and cardiac citrate levels increases up to 11-fold as compared to sham [17]. Gleaves and colleagues reported a patient with DFE inhalation diagnosed with refractory hypocalcemia requiring 36 grams of calcium gluconate intravenously (IV), 2 grams of calcium chloride IV, and 5.6 grams of calcium carbonate by mouth for correction over the course of nine days [18]. Of note, fluoroethanes have been reported in the literature to be nontoxic or of low toxicity when administered via non-inhalational routes, suggesting that this particular cause of hypocalcemia is unique to inhalant use [17].

Hypocalcemia is known to precipitate cardiac arrhythmias as cardiac calcium ion channels remain open for longer periods. This results in alteration of the slopes in phases 0 and 4 of the cardiac pacemaker cell action potential and slopes of phases 0, 1, and 2 of the cardiac myocyte action potential [19]. In a patient with pre-existing hypocalcemia and myocardial “sensitization” due to inhalant use as previously discussed, a calcium channel blocker may provoke arrhythmias and/or cause vasodilation leading to hypotension. In general, beta-blockers are preferred for the treatment of arrhythmias secondary to inhalant toxicity. Given this patient’s rapid improvement with spontaneous conversion to normal sinus rhythm in conjunction with the relatively short half-life of DFE, supportive care alone may be the best option for these patients, particularly in those with lower overall amounts of adipose tissue [20,21].

## Conclusions

This case elucidates a rare presentation of a young patient with paroxysmal atrial fibrillation with a rapid ventricular response and severe hypocalcemia. This case offers a unique opportunity to explore the clinical presentation of the combination of the two pathological pathways associated with inhalant use. Given the increasing incidence of inhalant use among youths and adolescents, physicians should consider the combination of these two pathophysiological pathways for the clinical management of patients.
